# Supplementary material for: Development of a Polymersome-Based Nanomedicine for Chemotherapeutic and Sonodynamic Combination Therapy
Source: Int J Mol Sci. 2023 Jan 7;24(2):1194. doi: 10.3390/ijms24021194 (PMC9864053; doi:10.3390/ijms24021194)
Supplement: Supplementary file 1 [file ijms-24-01194-s001.zip › ijms-2134807-supplementary.pdf]

Supplementary Materials

Development of a Polymersome-Based Nanomedicine for Chemotherapeutic and Sonodynamic Combination Therapy

Mingyun Kim [1,†], Doyeon Kim [1,†], Yongho Jang [1], Hyounkoo Han [1], Seonock Lee [2], Hyungwon Moon [3], Jungho Kim [2] and Hyuncheol Kim [1,*]

[1] Department of Chemical and Biomolecular Engineering, Sogang University, 35 Baekbeom-ro, Mapo-gu, Seoul 04107, Republic of Korea
[2] Department of Life Science, Sogang University, 35 Baekbeom-ro, Mapo-gu, Seoul 04107, Republic of Korea
[3] R&D Center, IMGT Co., Ltd., Seongnam-si, Gyeonggi-do 13605, Republic of Korea
* Correspondence: hyuncheol@u.sogang.ac.kr
† These authors contributed equally to this work.

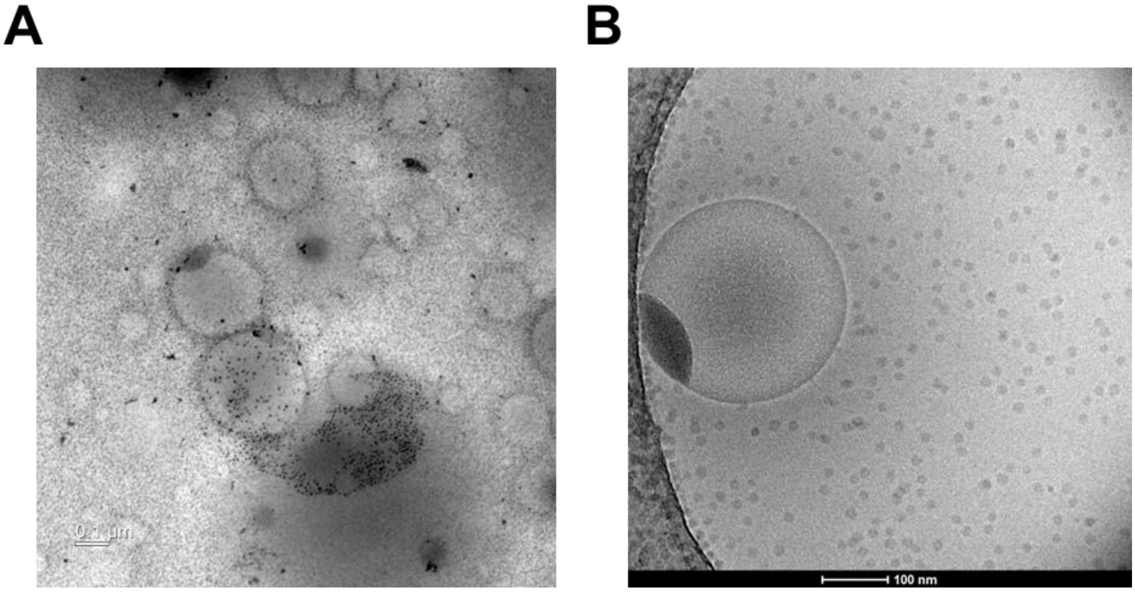

Figure S1. (A) TEM images of VP-DOX-PLs using gold nanoparticles (B) Cryo-EM image of VP-DOX-PLs

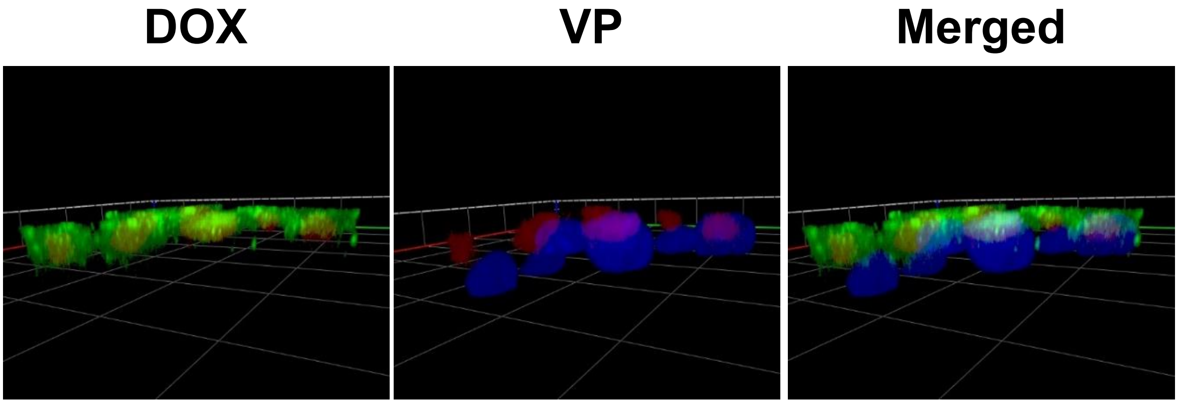

Figure S2. 3D stacking images of VP-DOX-PLs. (40x magnification)

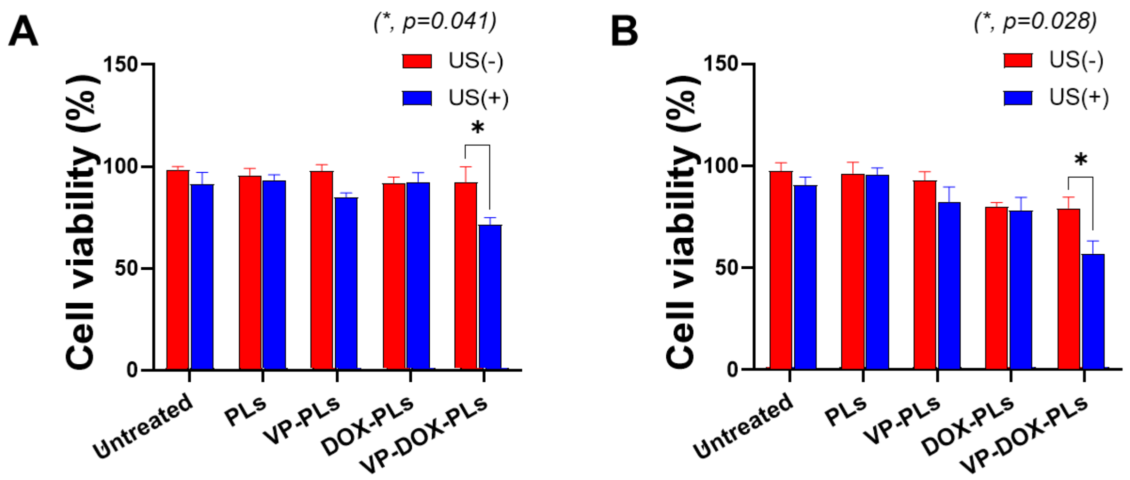

Figure S3. Cytotoxicity of VP-DOX-PLs compared with other groups with and without US. US was applied with a power of $0.3 W/cm^2$ and duty cycle of 50% for 10 seconds per well. Asterisk (*) means P value less than 0.001 compared with all other groups (P<0.001) after (A) 3h and (B) 6h

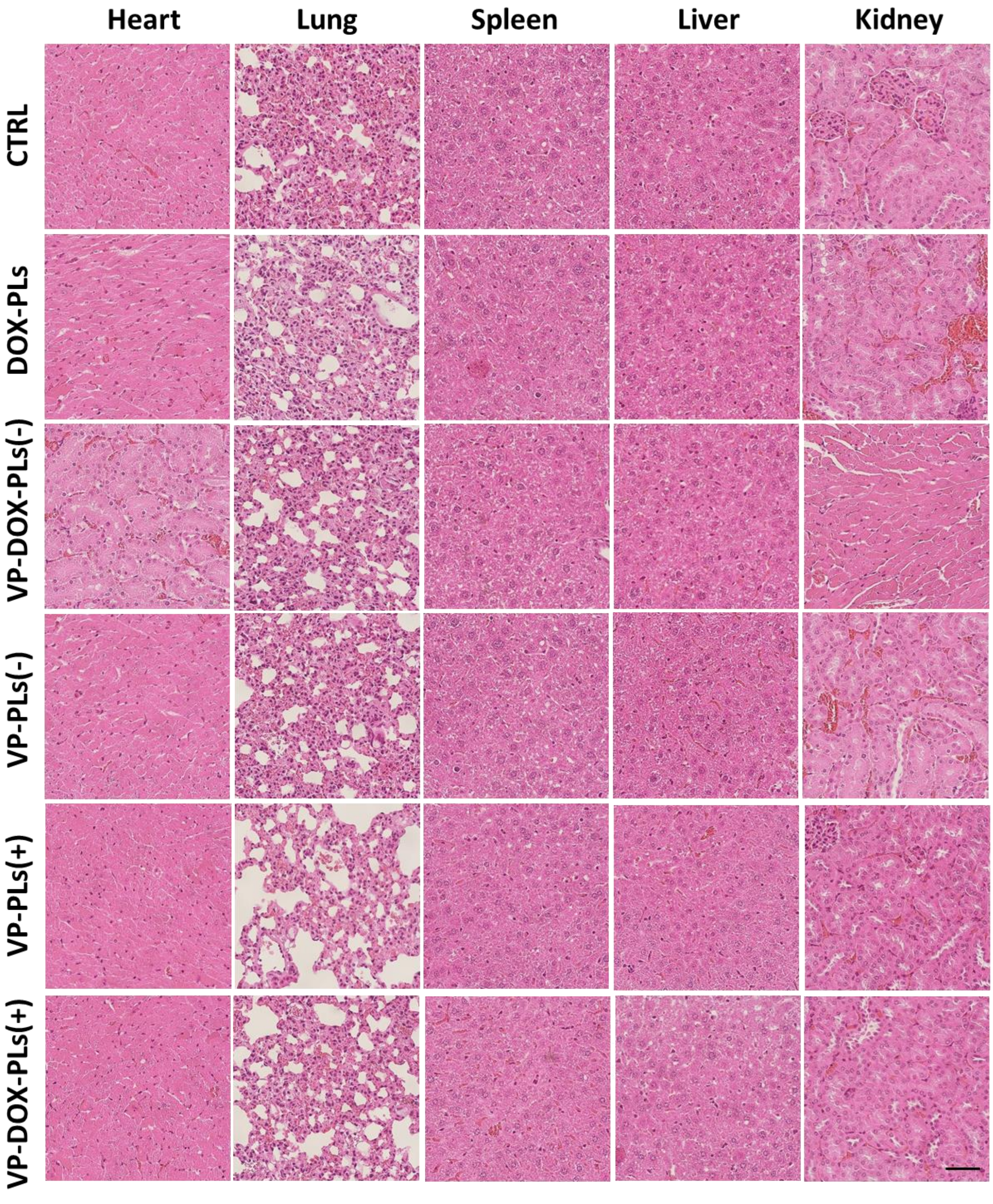

Figure S4. Histopathological examination of various treatment groups. H&E staining of normal organs from tumor-bearing nude mice treated with various treatment groups. Scale bar indicates 40 μm.
